# Auxin Directly Upregulates *GhRAC13* Expression to Promote the Onset of Secondary Cell Wall Deposition in Cotton Fibers

**DOI:** 10.3389/fpls.2020.581983

**Published:** 2020-11-05

**Authors:** Mi Zhang, Huizhen Cao, Jing Xi, Jianyan Zeng, Juan Huang, Baoxia Li, Shuiqing Song, Juan Zhao, Yan Pei

**Affiliations:** ^1^Biotechnology Research Center, Southwest University, Chongqing, China; ^2^Chongqing Key Laboratory of Plant Resource Conservation and Germplasm Innovation, Southwest University, Chongqing, China

**Keywords:** auxin, cellulose deposition, cotton fibers, transition, GhRAC13

## Abstract

Cotton fibers are single cells that show a relatively independent developmental process of cell differentiation, elongation, and secondary wall deposition. Auxin promotes fiber cell protrusion from the surface of the ovule. However, the role of auxin at other stages of cotton fiber development remains largely unknown. To gain a deeper insight into this aspect, we measured indoleacetic acid (IAA) content in developing fibers. Results showed an increase in IAA content at the transition stage from elongation to secondary cell wall deposition. Subsequently, we investigated the differences between two transgenic cottons that show upregulated and downregulated fiber auxin levels, respectively. *In planta* analysis revealed that, in addition to promoting cell elongation, auxin regulated the time of initiation of reactive oxygen species (ROS) production and secondary wall deposition in cotton fibers. This was closely correlated with the upregulated expression of *GhRAC13*, which regulates ROS-triggered cellulose synthesis. We found multiple putative auxin-responsive elements existed within the promoter region of *GhRAC13*, and IAA could induce *proGhRAC13* activity. The dual-luciferase reporter assay further proved the activation of *proGhRAC13* by GhARF5, an auxin-signaling activator. Altogether, our results suggest a role of auxin in promoting the onset of secondary growth by directly upregulating *GhRAC13* expression in cotton fibers.

## Introduction

Cellulose is a linear polysaccharide polymer that provides plants with strength and flexibility. Cellulosic biomass accounts for the largest proportion of plant cell wall components, which can be used as renewable biofuels. In particular, over 90% of the cotton fiber biomass is pure cellulose (Meinert and Delmer, 1977), making it the major source of natural fiber worldwide. Further, given their single-cell structure, cotton fibers provide an excellent system to study the mechanism and regulation of cellulose synthesis (Haigler et al., 2012).

The development of cotton fiber includes four consecutive but partially overlapping stages: initiation, elongation, secondary wall deposition, and maturation. During fiber development, cellulose is massively synthesized and deposited into the secondary wall in the third stage. At the transition stage from elongation to secondary wall deposition, which normally occurs approximately 16 days post-anthesis (DPA), strong depletion of carbohydrates is coupled with a sharp increase in cellulose synthesis (Meinert and Delmer, 1977; Zhang et al., 2012) and the secondary cell wall appears highly crystalline (Basra and Saha, 1999). Ultimately, the secondary wall reaches 8–10 μm in thickness inside the primary wall, the thickness of which is normally 0.2–0.4 μm (Wilkins and Arpat, 2005). Cellulose synthases (CesAs) are the main enzyme complexes responsible for cellulose synthesis. Among them, CesA4/7/8 plays a key role in secondary wall synthesis (Kumar et al., 2017). In cotton, *celA1* (a homolog to AtCesA8) and *celA2* (a homolog to AtCesA4) are highly expressed in correlation to secondary wall-associated cellulose synthesis (Pear et al., 1996; Kim and Triplett, 2007). Subsequently, other members of this protein family have been analyzed. Homologs to CesA4/7/8 are shown to have a similar expression pattern in developing fibers during secondary wall deposition (Kim and Triplett, 2007; Niu et al., 2016). In addition to CesAs, actin-associated proteins GhADF (Wang et al., 2009) and GhPFN2 (Wang et al., 2010) as well as the receptor-like kinase GhRLK (Li et al., 2005) also play important roles in secondary cell wall formation. The reactive oxygen species (ROS) hydrogen peroxide (H_2_O_2_) is a signaling molecule that controls the differentiation of secondary wall during fiber development (Potikha et al., 1999). Further, the generation of H_2_O_2_ is mediated by GhRAC13, a small GTP-binding protein preferentially expressed in developing cotton fibers from 14 to 24 DPA (Delmer et al., 1995). Consistently, ectopic expression of GhRAC13 reportedly increases H_2_O_2_ levels in soybean and *Arabidopsis* cells (Potikha et al., 1999).

Plant hormones are pivotal regulators involved in cotton fiber development. For example, fiber cell initiation is influenced by auxin (Zhang et al., 2011), gibberellic acid (Xiao et al., 2010), cytokinins (Zeng et al., 2012), brassinosteroids (Luo et al., 2007), and jasmonic acid (Hu et al., 2016); in turn, fiber cell elongation responds to auxin (Gokani and Thaker, 2002), ethylene (Shi et al., 2006), and gibberellic acid (Xiao et al., 2010). Finally, secondary wall deposition is seemingly influenced by abscisic acid (Yang et al., 2001). Previously, we observed auxin accumulation in initiating fiber cells, of which the accumulation is possibly mediated by delocalization of auxin efflux carrier GhPIN3a (Zhang et al., 2011, 2017; Zeng et al., 2019). Increasing level of indoleacetic acid (IAA) in the ovule epidermis increases fiber number and results in fine fibers (Zhang et al., 2011). Therefore, we hypothesized that auxin plays other roles in fiber development in addition to promoting fiber initiation. Determination of plant hormone levels indicated that the level of IAA in elongating fibers will rise again as the time for secondary cell wall deposition nears (Nayyar et al., 1989; John, 1999; Gokani and Thaker, 2002), thus suggesting a possible involvement of auxin at this point. This agrees with results of a study on auxin metabolism, which reported higher IAA catabolism during secondary cell wall deposition than during cell elongation (Jasdanwala et al., 1977). Moreover, naphthalene-1-acetic acid-induced *in vivo* inhibition of cellulose synthesis in cotton fibers also suggests that auxin may influence secondary growth of cotton fibers (Singh et al., 2009). However, the exact role of auxin in other fiber developmental stages is still a mystery. Therefore, in this study, we used two transgenic cotton genotypes with increased or decreased auxin level in developing fibers for investigating the role of auxin in secondary cell wall deposition.

## Materials and Methods

### Plant Materials and Transformation

Wild-type *Gossypium hirsutum* ‘Jimian 14’ and transgenic cotton genotypes *SCFP:iaaM* and *DR5:GUS* have been described previously (Zhang et al., 2011, 2017). The coding sequence of *iaaL* was amplified with flanking sites *Xba*I and *Eco*RI to replace *iaaM* for generating the *SCFP:iaaL* construct. Cotton transformation was performed using an *Agrobacterium*-mediated method previously reported (Luo et al., 2007). After selection by kanamycin and β-glucuronidase (GUS) staining, positive plantlets were transferred to a glasshouse. The homogenic lines were planted in the experimental field in the summer for further observation. Mature fibers (>10 g) were sent to the Center of Cotton Fiber Quality Inspection and Testing, Chinese Ministry of Agriculture (Anyang, Henan Province, China) for quality measurement.

### Histochemical Staining and Quantification of GUS Activity

Histochemical staining was performed according to a previously described method (Zhang et al., 2017). After excision, ovules with fibers were stained in the dark at 37°C for 12 h. After staining, samples were kept in 75% ethanol for observation. Fluorometric assay of GUS activity was performed using a previously reported method (Hou et al., 2008). Fresh fibers were ground in liquid nitrogen and homogenized in GUS extracting buffer [50 mM phosphate buffer solution (PBS) (pH 7.0), 100 mg/ml PVP, and 10 mM β-met]. After incubation on ice for 1 h, the extract was centrifuged at 13,000 rpm for 10 min. The supernatant was the total protein crude extract. Protein concentration was determined by the Bradford method. GUS activity was calculated as pmol 4-MU mg^–1^ protein min^–1^, and each test was performed with three biological replicates.

### Extraction and Determination of IAA

IAA extraction was performed according to the method described by Zhang et al. (2011). Approximately 200 mg fresh weight of cotton fiber was homogenized in liquid nitrogen, then extracted overnight at 4°C with 7 ml of 80% methanol containing 10 ng ^13^C_6_-IAA as an internal standard. After centrifugation for 20 min at 10,000 × *g* at 4°C, the supernatant was collected and evaporated under vacuum at 40°C. The residue was dissolved in 0.1 M acetic acid and applied to a pre-equilibrated Sep-Pak^®^ Plus tC18 cartridge (Waters, United States). Retained IAA was first washed with 4 ml of 17% methanol, then eluted with 6 ml of 40% methanol, and finally dried under vacuum. Each sample was redissolved in 10% methanol, filtered through a 0.22-μm filter, and analyzed on a 4000Q TRAP LC/MS/MS system (ABsciex, United States) as previously described (Zeng et al., 2019).

### Quantitative PCR Analysis

Approximately 1 g cotton fiber was used for RNA extraction with the easy-spin Plant RNA Extraction Kit (Aidlab, China). cDNA synthesis and gene expression were analyzed according to a method previously described (Zhang et al., 2017). Forward and reverse primers used are listed in Supplementary Table 1. *GhHIS3* (Gohir.D03G040300) and *AtACT2* (AT3G18780) were used as reference genes in cotton and *Arabidopsis*, respectively. Each experiment was performed at least three times showing the same expression profile consistently, whereby only one result is shown here.

### Ovule Culture and Measurement of Fiber Length

Ovule culture was performed as describe before (Zhang et al., 2017). Ovules at 1 DPA were floated on the BT medium (Beasley, 1973) supplemented with IAA (at concentrations ranging from 0.5 to 50 μM) and 0.5 μM GA_3_. After a 2-week cultivation, ovules were observed under an MVX10 stereomicroscope (Olympus). For measurement of fiber length, ovules attached with fibers were boiled in water for 10 min. Then, fibers were combed for measurement and observation. Twenty ovules were used for each treatment.

### Visualization of Crystalline Cellulose

Cotton fibers were separated at different times and mounted in distilled water. Crystalline cellulose was observed by birefringence microscopy (Potikha et al., 1999) using an IX81 inverted microscope system (Olympus).

### Determination of Cellulose Content

The assay was carried out using a previously described method (Updegraff, 1969), after some modifications. Cotton fibers were ground in liquid nitrogen. Homogenized samples (∼0.2 g each) were suspended in 5 ml acetic/nitric solution (acetic acid/distilled water/nitric acid = 4:1:5) and heated in boiling water for 30 min. The extract was then centrifuged at 3,000 × *g* for 10 min, and the supernatant was discarded. The pellet was washed by suspending and pelleting three times in 8 ml of distilled water. Then, the residue was suspended in 5 ml 80% H_2_SO_4_ (*v*/*v*) and incubated at room temperature for 1 h. A 100-μl sample was mixed with ice-cold anthrone reagent [0.2 g anthrone dissolved in 100 ml 80% H_2_SO_4_ (*v*/*v*)]. After incubation at room temperature for 10 min, optical density at 620 nm was recorded on an infinite M200PRO (TECAN) plate reader. The concentration of cellulose was determined according to a standard curve obtained using pure cellulose.

### ROS Visualization

To obtain a 10-mM stock solution, 2′,7′-dichlorodihydrofluorescein diacetate (H_2_DCFDA AM; Sigma, United States) was dissolved in DMSO. Ovules attached to fibers were washed three times in 10 mM PBS (pH 5.2). Then, they were immersed in H_2_DCFDA working solution (diluted in 10 mM PBS pH 5.2 at 1:1,000) for 10 min at 25°C in darkness. After staining, samples were washed again six times with 10 mM PBS (pH 5.2). The fluorescent signal of excitation at 488 nm and the emission spectrum over 500–550 nm were observed using an IX81 inverted microscope system (Olympus).

### Activity Assay of the GhRAC13 Promoter

To analyze the inducible activity of the GhRAC13 promoter, the upstream sequence of the *GhRAC13* (Gohir.D10G029600) coding region was amplified from upland cotton. The promoter flanked by sites *Hin*dIII and *Sma*I was placed in front of the β*-*glucuronidase (GUS) reporter gene in vector pBI121. The construct was then introduced into *Arabidopsis* with the mediation of *Agrobacterium* (strain GV3101) by the floral dip method (Clough, 2004). For dual-luciferase reporter assay, the promoter sequence of *GhRAC13* was placed upstream of *LUC* gene in expression vector pGreen0080 using the homologous recombination method; the expression vector pGreen0080 harbored another cassette of *Renilla* luciferase driven by 35S promoter. *GhARF5* (Gohir.A05G187900) was amplified from cDNA of elongating cotton fibers and cloned into a modified vector pLGN under the control of 35S promoter (Zeng et al., 2019). Both constructs were transformed into *Agrobacterium* (strain GV3101) and transiently expressed in *Nicotiana benthamiana* leaves. Three days later, infiltrated leaves were used for the dual-luciferase reporter assay using Dual-Glo^®^ Luciferase Assay System (Promega, United States).

## Results

### Auxin Content in Developing Cotton Fibers

**FIGURE 1:**
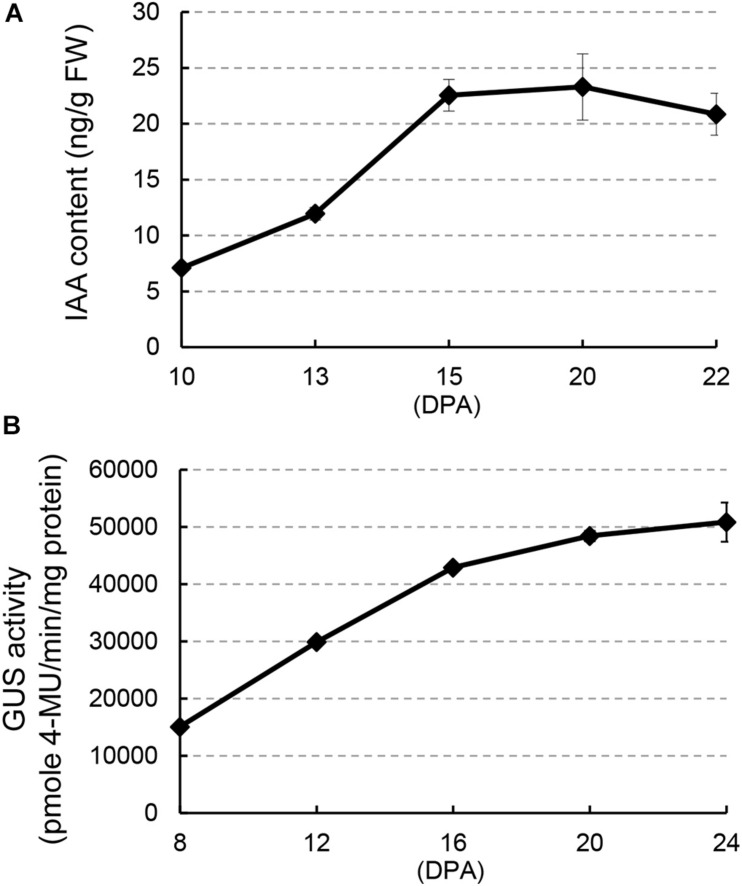
Auxin level increases in fiber cells during the transition between elongation and cell wall secondary growth onset. **(A)** Free indoleacetic acid (IAA) content in developing fibers at different days post-anthesis (DPA). **(B)** GUS activity in developing fibers of *DR5:GUS* cotton. Error bars indicate standard deviation of three biological replicates.

To investigate a possible regulatory role of auxin during cotton fiber development stages, we analyzed IAA content in developing fibers of upland cotton (*Gossypium hirsutum* ‘Jimian 14’) from 10 to 22 DPA (Figure 1A), which comprised fiber development stages from elongation to early secondary wall deposition. The IAA level increased remarkably (over two-fold) from 10 to 15 DPA and thereafter remained at a relatively high level until 22 DPA. GUS activity assay in *DR5:GUS* (β*-glucuronidase*) cotton, an auxin reporter system, confirmed this result: strong GUS staining was detected in fibers (Supplementary Figure 1). In addition, quantitative analysis of GUS activity showed a considerable increase from 8 to 16 DPA (Figure 1B). Both results showed an increase in IAA content at approximately 15 DPA, suggesting a possible auxin-mediated regulation of fiber development at this stage.

### Changes in Endogenous IAA Content in Transgenic Cotton Fibers

**FIGURE 2:**
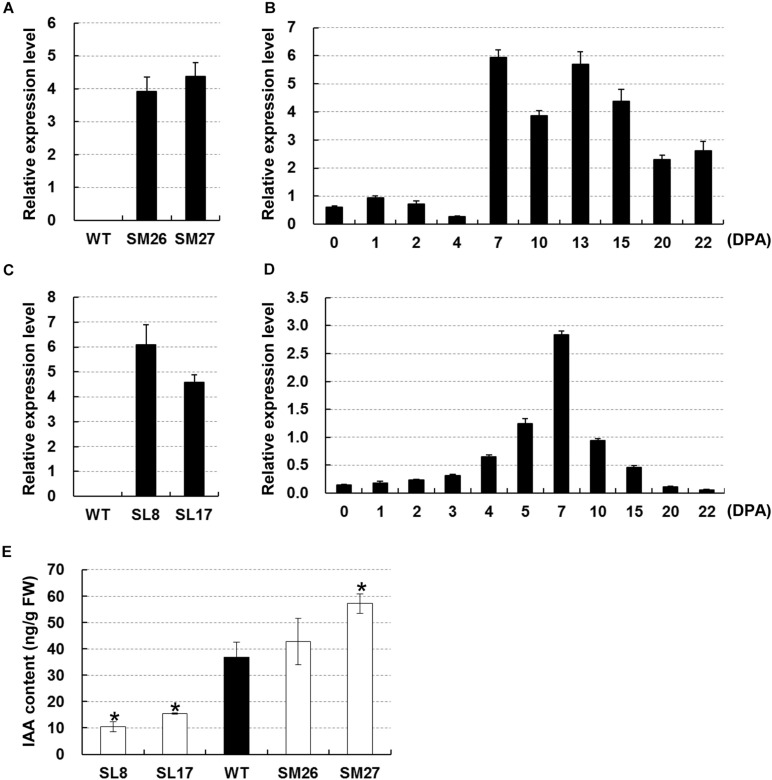
Manipulation of auxin level in cotton fibers. **(A)** Expression of *iaaM* in 15-DPA fibers of *SCFP:iaaM* cotton. **(B)** Expression of *iaaM* in developing ovules and fibers of *SCFP:iaaM* cotton. **(C)** Expression of *iaaL* in 15-DPA fibers of *SCFP:iaaL* cotton. **(D)** Expression of *iaaL* in developing ovules and fibers of *SCFP:iaaL* cotton. **(E)** Free indole acetic acid (IAA) content in wild-type and transgenic cotton fibers at 15 DPA. Error bars indicate standard deviation of three biological replicates. Wildtype in **(A)** and **(C)** was used as negative control. SM, *SCFP:iaaM* transgenic cotton; SL, *SCFP:iaaL* transgenic cotton; WT, wild-type; DPA, day post-anthesis. Asterisks indicate significant difference as determined by Student’s *t*-test (*P* < 0.05).

We used two transgenic cottons, namely, *SCFP:iaaM* and *SCFP:iaaL*, both with modified auxin content in fibers, to test the role of auxin during early secondary wall deposition. Auxin biosynthesis-related gene *iaaM* and metabolism-related gene *iaaL* (Glass and Kosuge, 1986) were driven by a fiber-specific promoter *SCFP*. Two independent *SCFP:iaaM* lines (#26 and #27) were screened based on the transcription of *iaaM* in fibers at 15 DPA (Figure 2A). The transcription data showed that *iaaM* was highly expressed in fibers between 7 and 22 DPA (Figure 2B), resulting in a 16% and a 55% increase in IAA content in fibers of lines #26 and #27, respectively, compared with that in the wild-type (Figure 2E). Likely, we selected two lines with high expression of *iaaL* in fibers at 15 DPA (Figure 2C). The *iaaL* transcript was detected at high levels in fast elongating fibers from 4 to 15 DPA (Figure 2D). Measurements of IAA showed that fiber IAA content was 10.39 and 15.37 ng/g in lines SL8 and SL17 at 15 DPA, respectively, i.e., 72% and 58% lower than that in the wild-type (Figure 2E). As expected, all transgenic cotton lines showed modified fiber IAA content and were used for further assays.

### Expression of Development-Related Genes in Transgenic Cotton Fibers

**FIGURE 3:**
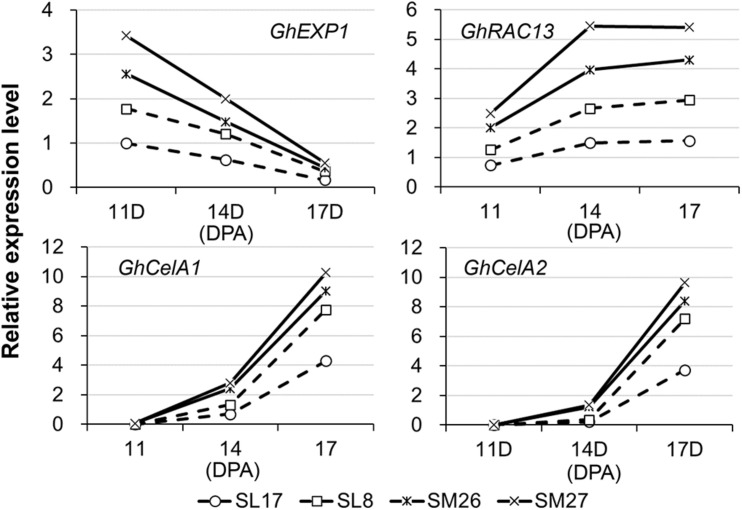
Expression of cell elongation- and cellulose synthesis-related genes in developing fibers. SM, *SCFP:iaaM* transgenic cotton; SL, *SCFP:iaaL* transgenic cotton; DPA, day post-anthesis.

To examine what specific event during fiber development is affected by auxin, we selected four marker genes related to elongation (*GhEXP1*) and secondary wall deposition (*GhRAC13*, *GhCESA1*, and *GhCESA2*) (Singh et al., 2009) and analyzed the corresponding level of transcription in fibers (Figure 3). Developing fibers showed a decreasing level of *GhEXP1* transcript, while an increasing level of *GhRAC13*, *GhCESA1*, and *GhCESA2* transcripts from 11 to 17 DPA. We thus inferred that fiber elongation slows, while secondary wall thickening begins at this time. All the four genes were remarkably upregulated in fibers expressing *iaaM*, particularly *GhEXP1* at 11 DPA and *GhRAC13*, *GhCESA1*, and *GhCESA2* after 14 DPA. This finding suggested that both fiber elongation and secondary wall deposition are promoted in *iaaM*-expressing fibers, in which IAA content was enhanced.

### Auxin Promotes Fiber Cell Elongation

**FIGURE 4:**
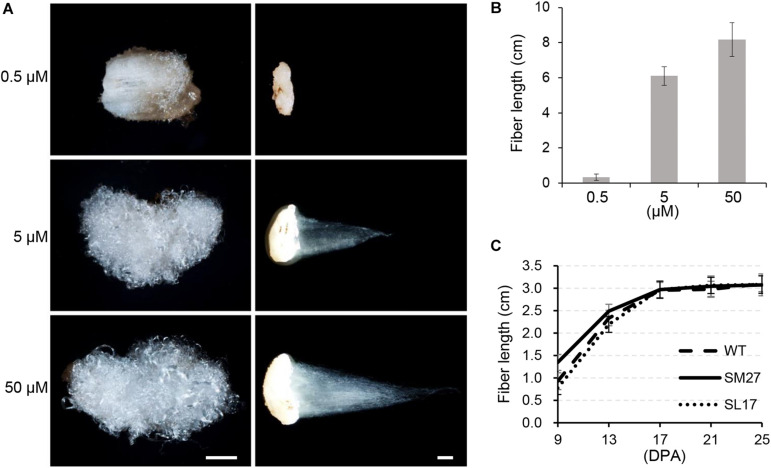
Auxin promotes fiber elongation. **(A)** Indoleacetic acid (IAA) treatment increases fiber length *in vitro*. Scale bar = 1 mm. **(B)** Fiber length under IAA treatment *in vitro*. **(C)** Fiber length of transgenic cotton. SM, *SCFP:iaaM* transgenic cotton; SL, *SCFP:iaaL* transgenic cotton. Error bars indicate standard deviation (*n* ≥ 10).

When we measured mature fiber length to evaluate the effect of auxin on fiber elongation, we found no obvious difference in fiber length between transgenic and wild-type cotton genotypes (Supplementary Table 2). We suspected that the effect of the modified IAA level on fiber elongation might be too slight to be quantified. Therefore, we investigated the *in vitro* effect of IAA using cultured ovules harvested at 0 DPA, in which fiber cells already appear. Two weeks later, fiber length on cultured ovules was increased with increasing IAA supplement from 0 to 50 μM (Figures 4A,B). We then determined the length of developing fibers in transgenic cotton lines (Figure 4C). The results showed that *SCFP:iaaM* fibers were longer than *SCFP:iaaL* fibers during the elongation stage (9 to 17 DPA). However, since then, the elongation rate of fibers decreased more strikingly in the *SCFP:iaaM* than in the *SCFP:iaaL* cotton line, resulting in a similar fiber length afterward (starting at 17 DPA). These results suggested that the upregulation of IAA level could promote fiber cell elongation, but the effect might have been gradually attenuated subsequently.

### Auxin Activates Cellulose Synthesis for Secondary Wall Deposition

**FIGURE 5:**
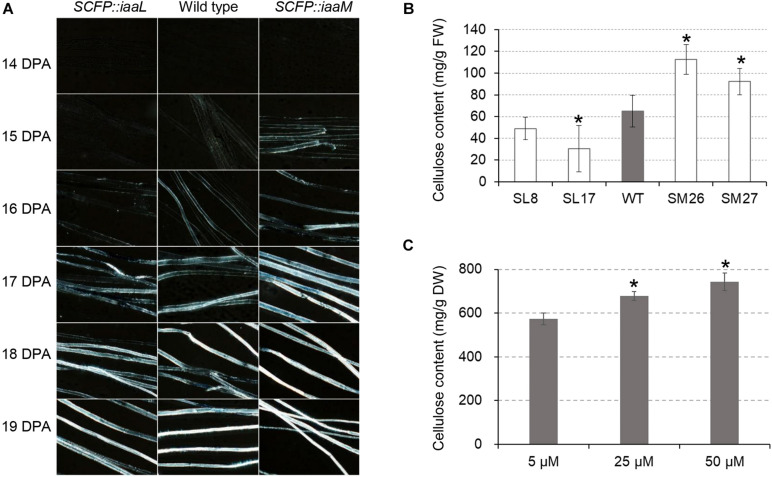
Auxin promotes the onset of cellulose deposition in cotton fibers. **(A)** Cellulose deposition in developing fibers. **(B)** Cellulose content in cotton fibers at 15 days post-anthesis (DPA). **(C)** Cellulose content in IAA-treated fibers. Ovules (0 DPA) were cultured *in vitro* for 30 days. WT, wild-type; SM, *SCFP:iaaM* transgenic cotton; SL, *SCFP:iaaL* transgenic cotton. Error bars indicate standard deviation based on three biological replicates. Asterisks indicate significant difference (vs. wild-type or 5 μM) as determined by Student’s *t*-test (*P* < 0.05).

We investigated the role of auxin in secondary wall deposition through the observation of crystalline cellulose in developing fibers. Using polarization microscopy to observe birefringence of crystalline cellulose in cells, we examined cellulose synthesis in transgenic and wild-type cotton fibers (Figure 5A). The white signal of birefringence was first observed in the IAA-increased fibers (*SCFP:iaaM*) at 15 DPA, becoming stronger thereafter. A similar signal pattern appeared at 16 DPA in wild-type fibers, while in the IAA-decreased fibers (*SCFP:iaaL*), it appeared at 17 DPA. When determining fiber cellulose content at 15 DPA, we found a pronounced increase in *SCFP:iaaM* fibers and a decrease in *SCFP:iaaL* fibers relative to the wild-type (Figure 5B). *In vitro* culture in the presence of different IAA concentrations confirmed the stimulating effect of IAA on cellulose synthesis (Figure 5C). The change in cellulose synthesis was expected to increase fiber fineness in transgenic cotton. Surprisingly, however, micronaire values, which provide an evaluation of both fineness and maturity of cotton fibers, slightly increased in *SCFP:iaaL* and decreased in *SCFP:iaaM* fibers compared with wild-type fibers (Supplementary Table 2). Therefore, we hypothesized that auxin only activates the onset of cellulose synthesis rather than continuously enhances cellulose synthesis.

### Auxin Stimulates ROS Production

**FIGURE 6:**
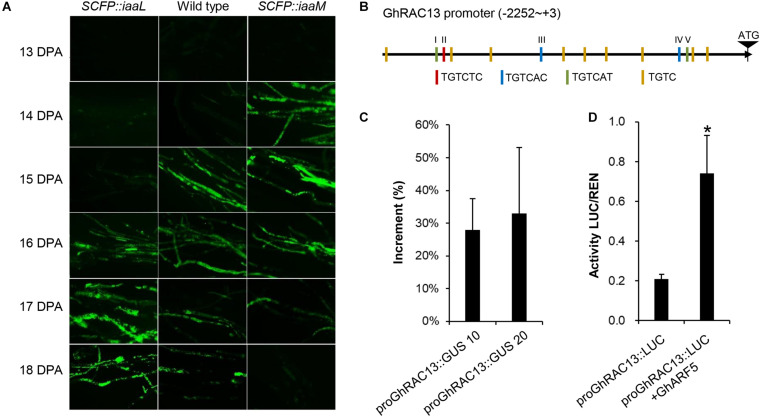
Auxin causes earlier occurrence of GhRAC13-mediated ROS generation in cotton fibers. **(A)** ROS generation occurred earlier in transgenic fibers with higher indoleacetic acid (IAA) levels. **(B)** Diagram of AuxREs in *GhRAC13* promoter. **(C)** Activity of *GhRAC13* promoter was upregulated by IAA treatment. **(D)** *GhRAC13* promoter was activated under the expression of GhARF5. Error bars indicate standard deviation based on three biological replicates. Asterisks indicate significant difference as determined by Student’s *t*-test (*P* < 0.05).

A previous study revealed that ROS generation is a crucial signal for triggering cellulose synthesis during secondary wall deposition of cotton fibers. Hence, we analyzed ROS production in developing fibers using fluorescent dye H_2_DCFDA AM (Figure 6A). A large amount of fluorescent signal indicating active ROS generation appeared first at 15 DPA in wild-type cotton fibers, remaining strong until 16 DPA and decreasing sharply after 18 DPA. This finding clearly indicated a ROS burst in developing cotton fibers from 15 to 18 DPA. In contrast, the first appearance of the ROS signal in IAA-increased fibers (*SCFP:iaaM*) was at 14 DPA, 1 day earlier than in the wild-type. Meanwhile in IAA-decreased fibers (S*CFP:iaaL*), ROS generation started at 16 DPA, 1 day later than in the wild-type. These results suggested that the ROS-signaling pathway could be activated earlier in fibers at the transition stage when intracellular IAA level was upregulated, and the pathway could be delayed when the level was downregulated.

### *GhRAC13* Expression Is Upregulated by Auxin Signaling

The earlier generation of ROS, coupled with the upregulated expression of *GhRAC13* in *SCFP*:*iaaM* fibers, prompted us to verify whether IAA transcriptionally regulates *GhRAC13*. Thus, we amplified the ∼2.3-kb upstream regulatory region of *GhRAC13* in the D subgenome. In the promoter sequence, we identified five putative auxin response elements (AuxREs) and the other nine variants containing the core motif TGTC (Figure 6B) based on the report by Sghaier et al. (2018), indicating that auxin signaling may directly regulate *GhRAC13* transcription. We then fused the promoter to the *GUS* reporter gene and transformed it into *Arabidopsis*. Due to the preferential expression of *GhRAC13* in fibers at the transition stage (Delmer et al., 1995), histochemical staining was not sensitive enough to detect the activity of the promoter in transgenic leaves. Alternatively, qRT-PCR was used to quantify the expression of the *GUS* gene. When leaves were treated with 20 μM IAA for 4 h, *GUS* expression was considerably upregulated by 18% to 33% (Figure 6C). We further used the dual-luciferase reporter assay to confirm this result. ARF5 is a canonical transcriptional activator in the auxin-signaling pathway (Guilfoyle and Hagen, 2007). Under co-expression with *GhARF5* (Gohir.A05G187900), a homolog of *ARF5* expressed in fibers at the transition stage, *LUC* driven by *proGhRAC13* was significantly upregulated (Figure 6D). These results indicated that auxin directly regulates the expression of GhRAC13 through the auxin signaling pathway.

## Discussion

Studies on the roles of auxin in cotton fiber development had been mainly limited to the stage of cell initiation (Beasley, 1973; Gialvalis and Seagull, 2001; Seagull and Giavalis, 2004; Zhang et al., 2011). In the present study, the results of *in vitro* experiments using transgenic cotton fibers suggest a promotive role of auxin in cell elongation (Figure 4), which is analogous to previous observations *in vitro* (Gokani and Thaker, 2002) and studies revealing polar growth in most cells (Cleland, 2007). Here, we clearly showed that auxin regulates the onset of secondary cell wall deposition as well. Consistent to previous studies (Nayyar et al., 1989; John, 1999; Gokani and Thaker, 2002), an increase of auxin level was found in fibers of the transition stage (Figure 1). Auxin biosynthetic pathway is not remarkably upregulated in developing fibers (Shi et al., 2006). We recently revealed that, instead of local synthesis, auxin corresponding to fiber initiation is mainly imported from the outside of ovules, and the asymmetry localization of the auxin efflux carrier GhPIN3a plays a pivotal role for the specific accumulation of IAA in fiber cells (Zhang et al., 2017; Zeng et al., 2019). The increase of auxin level was coupled with the decrease of cell expansion or elongation (Figure 4C) (Gokani and Thaker, 2002). However, this does not disagree the promotive effect of auxin on fiber elongation. Instead, the increased auxin level upregulated the expression of *GhRAC13* (Figures 3, 6C), which controls ROS production in cells (Potikha et al., 1999), thereby triggering the onset of secondary growth (Potikha et al., 1999); the cell wall rigidity given by the secondary growth consequently slows down fiber elongation. Additionally, our study indicated that ROS production occurred approximately 1 day earlier than the observation of crystalline cellulose (Figures 5A, 6A). Subsequently, cellulose synthesis indicated by increased *GhCelA1/2* expression was promoted (Figure 3) and much crystalline cellulose was deposited into the secondary cell wall (Figure 5A). Altogether, our data have revealed a previously unknown role of auxin in controlling the developmental transition from elongation to cellulose deposition in cotton fibers.

GhRAC13, also named GhROP7, is a homolog of small GTP-binding proteins in upland cotton. The activation of GhRAC13 reportedly increases ROS level when overexpressed in leaves (Potikha et al., 1999). In this study, we revealed that upregulation of *GhRAC13* had a similar effect when induced by a high intracellular level of auxin (Figures 3, 6C). This possibly increased the amount of GhRAC13 protein to be activated. Moreover, our results further demonstrate that the expression of *GhRAC13* can be activated through at least GhARF5-mediated auxin signaling pathway (Figures 6B,D). This illustrates the role of auxin–GhRAC13–ROS regulation pathway in the transition of cotton fibers from the stage of elongation to the stage of secondary wall deposition.

We found no correlation between modified cell elongation or secondary growth onset and mature fiber quality. The ultimate fiber traits agree with previous studies on transgenic cottons overexpressing auxin biosynthesis-related genes in fibers (John, 1999; Zhang et al., 2011). However, as shown herein, the decrease in elongation rate demonstrates that auxin does not determine the ultimate fiber length (Figure 4C). Moreover, earlier cellulose deposition triggered by higher auxin levels does not lead to higher, but to slightly lower, micronaire value in transgenic cotton, indicating that fibers become finer as observed for *FBP7:iaaM* transgenic fibers (Zhang et al., 2011). A possible explanation is that the premature deposition of secondary cell wall increases the rigidity of fiber cells and thus limits cell expansion in length and diameter (Seagull et al., 2000). This limitation is weak in developing fibers with lower IAA level, although the initial elongation rate is lower as well (Figure 4C). Consequently, no change in the length of mature fibers was observed between IAA-increased and IAA-decreased fiber. Based on these findings, it is possible to genetically modify fiber length by combination strategies; for example, by increasing auxin level in fibers at the elongation stage and simultaneously deactivating GhRAC13 in fibers at the transition stage.

Our results indicate that auxin alters the timing of ROS production in fibers at the transition stage (Figure 6A) and that ROS production will be scavenged after the activation of cellulose synthesis. It remains unclear why the relatively high level of auxin in fiber cells between 15 and 22 DPA did not result in a continuous production of ROS through the auxin–GhRAC13–ROS pathway. The comprehensive regulation via the activation of GhRAC13 by ROP guanine nucleotide exchange factors and deactivation by ROP GTPase-activating proteins should be considered in future studies to address this question. Our results suggest a direct upregulation of GhRAC13 by the GhARF5-signaling pathway (Figure 6D); however, details regarding the precise mechanisms of this regulation warrant further exploration. The effect of auxin on decreasing micronaire value of mature fibers reported here has been indicated in previous transgenic cotton expressing *acsA* and *acsB* (Li et al., 2004), two genes involved in cellulose synthesis in *Acetobacter xylinum*. This implies a continuous upregulation of cellulose synthesis in cotton fibers overexpressing auxin. We conclude that the cause for low micronaire value might be the same as the one that contributed to the attenuation in fiber cell elongation: the increased cell wall rigidity limits the expansion in radial direction. However, whether auxin continues to upregulate cellulose synthesis after the initial activation of cellulose *via* the GhRAC13–ROS pathway, as well as the underlying mechanism of auxin’s effect on fiber fineness, needs further investigation.

Altogether, our results suggest a role of auxin in the transition stage through direct upregulation of *GhRAC13* expression, which in turn promotes the onset of ROS-triggered secondary growth in cotton fibers. This outlines an auxin–ROP–ROS–cellulose synthesis pathway in cell development, although details regarding this pathway need further exploration in order to improve our understanding of genetic modifications of cellulose synthesis in plants. Additionally, our study provides a basis for genetic improvement of fiber length through overexpressing auxin; however, we should develop an effective method that will prevent the inhibitory effect of auxin-induced onset of secondary wall deposition.

## Data Availability Statement

The original contributions presented in the study are included in the article/Supplementary Material, further inquiries can be directed to the corresponding author.

## Author Contributions

YP and MZ conceived the experiments and wrote the manuscript. HC, JX, JZe, JH, and BL performed the experiments and analyzed the data. SS and JZh analyzed the agronomic trait performance. All authors reviewed and approved the manuscript.

## Conflict of Interest

The authors declare that the research was conducted in the absence of any commercial or financial relationships that could be construed as a potential conflict of interest.
