# Supplementary material for: Auxin Directly Upregulates GhRAC13 Expression to Promote the Onset of Secondary Cell Wall Deposition in Cotton Fibers
Source: Front Plant Sci. 2020 Nov 5;11:581983. doi: 10.3389/fpls.2020.581983 (PMC7674626; doi:10.3389/fpls.2020.581983)
Supplement: Supplementary file 1 [file Data_Sheet_1.docx]

**
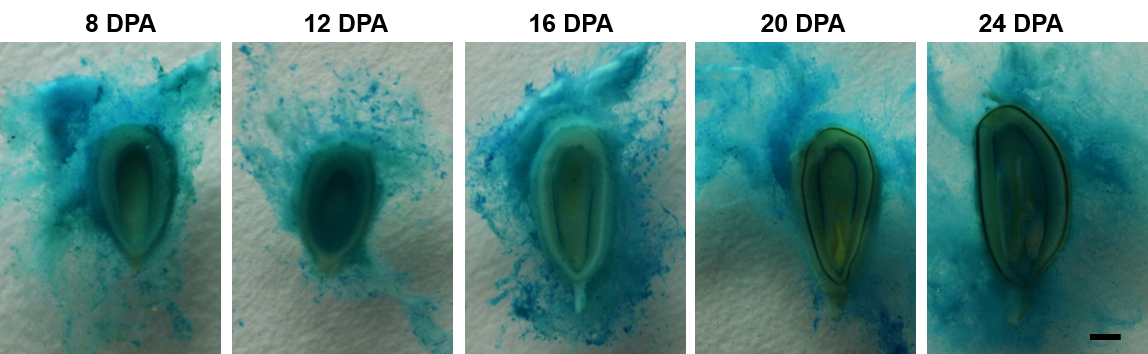
**

**Supplementary Figure 1** *DR5::GUS* expression level in developing ovules and fibers.

Ovules of *DR5::GUS* cotton were harvested and stained for 12h in the dark. Scale bar = 1 mm.

**Supplementary table 1** Primer sequences and their usage

| Gene Name | Forward primer (5’>3’) | Reverse primer (5’>3’) | Usage |
| --- | --- | --- | --- |
| *iaaM* | tttgcccagcacctatttc | aatagcaccctccacccat | qRT-PCR analysis |
| *iaaL* | gcgacaagctctacctgaca | gcctgaacgaaaacggtcac | qRT-PCR analysis |
| *celA1* | tggactacccggtggataaggt | ctttcttgcaaagtcggctgtt | qRT-PCR analysis |
| *celA2* | gcagcagacgatacagaattcg | cgttgttgattgcgtctgaaac | qRT-PCR analysis |
| *GhEXP1* | ccgtgacagccaccaacttt | ttctgctatccgcaagaatgc | qRT-PCR analysis |
| *GhRAC13* | gtgaaggctgttttcgatgct | tctccttttgcaaggctttctc | qRT-PCR analysis |
| *GhHIS3* | gaagcctcatcgataccgtc | ctaccactaccatcatggc | qRT-PCR analysis |
| *GUS* | ctgcgacgctcacaccgata | ttccagtaccttctctgccg | qRT-PCR analysis |
| *AtACT2* | gattcagatgcccagaagtcttg | tggattccagcagcttccat | qRT-PCR analysis |
| *proGhRAC13* | ctctgacaacctatacccaatccta | agttttccccacagctccat | Gene cloning |
| *GhARF5* | gagaggacagggtacggatccatgggttcctttgttgaagaga | aaagcagggaattctggtaccctacggctcattcttaggtgg | Gene cloning |

**Supplementary table 2** Fiber quality of transgenic cottons and wild type

| Line | Length (mm) | Uniformity % | Micronaire | Elongation rate % | Strength cN/tex |
| --- | --- | --- | --- | --- | --- |
| SL17 | 30.03±0.34 | 84.40±0.80 | 5.10±0.16 | 5.90±0.00 | 31.90±0.95 |
| SL8 | 28.63±0.34 | 83.37±0.80 | 4.99±0.16 | 6.20±0.00 | 30.03±0.95 |
| SM26 | 29.63±0.86 | 83.00±1.04 | 4.75±0.01 | 6.13±0.21 | 30.80±2.01 |
| SM27 | 30.01±0.06 | 82.33±0.21 | 4.56±0.22 | 6.03±0.06 | 30.40±1.18 |
| WT | 29.81±0.88 | 81.67±0.31 | 4.96±0.11 | 6.13±0.15 | 30.6±31.07 |

Each data is shown in mean±SD of three biological replicates.
